# Unconscious Cueing via the Superior Colliculi: Evidence from Searching for Onset and Color Targets

**DOI:** 10.3390/brainsci2010033

**Published:** 2012-02-15

**Authors:** Isabella Fuchs, Ulrich Ansorge

**Affiliations:** 1 Faculty of Psychology, University of Vienna, Liebiggasse 5, 1010 Vienna, Austria; E-Mail: isabella.fuchs@univie.ac.at; 2 Institute of Cognitive Science, University of Osnabrück, 49069 Osnabrück, Germany

**Keywords:** vision, attention, color, subliminal

## Abstract

According to the bottom-up theory of attention, unconscious abrupt onsets are highly salient and capture attention via the Superior Colliculi (SC). Crucially, abrupt onsets increase the perceived contrast. In line with the SC hypothesis, unconscious abrupt-onset cues capture attention regardless of the cue color when participants search for abrupt-onset targets (Experiment 1). Also, stronger cueing effects occur for higher than lower contrast cues (Experiment 2) and for temporally, rather than nasally, presented stimuli (Experiment 3). However, in line with the known color-insensitivity of the SC, the SC pathway is shunted and unconscious abrupt-onset cues no longer capture attention when the participants have to search for color-defined targets (Experiment 4) or color-singleton targets (Experiment 5). When using color change cues instead of abrupt-onset cues, the cueing effect also vanishes (Experiment 6). Together the results support the assumption that unconscious cues can capture attention in different ways, depending on the exact task of the participants, but that one way is attentional capture via the SC. The present findings also offer a reconciliation of conflicting results in the domain of unconscious attention.

## 1. Introduction

At any instance in time, our visual world provides a vast amount of visual input. Since human mental capacity is limited, only a fraction of the available information is selected for purposes such as perception, memory and action control, while the rest of the information is ignored. In the visual modality, this selectivity is called *selective visual attention* [1]. One important issue concerns the role of selective visual attention in the division of labor between unconscious (or subliminal) visual processing on the one hand and conscious vision on the other. A related important question is how this division of labor is implemented in the human brain’s visual system. 

One hypothesis about the relationship between conscious and unconscious vision is that selective visuo-spatial attention (*i.e.*, the selection of positions or areas in the visual field) is a necessary (though not sufficient [2]) prerequisite for at least some forms of conscious visual perception [3,4], but see [5]. For example, according to feature-integration theory, selective visuo-spatial attention is necessary for the binding of different visual features (e.g., red color and round shape) into one perceived object (e.g., a tomato) [6]. Crucially, if it is true that attention serves conscious visual perception, it follows that attention should (at least partly) operate before conscious vision—that is, visuo-spatial attention should operate on the basis of subliminal visual information, too [3].

This has been demonstrated in cueing experiments with abrupt-onset singleton cues [7,8]. In cueing experiments, participants have to search for targets, and in each trial, one target is shown at one out of several positions [9,10]. The participants have to find this target, discriminate it, and respond to it. When presenting a single abrupt-onset briefly before the target with a Stimulus Onset Asynchrony (SOA) of up to 200–300 ms at the same position (SP) as the target, locating the target is facilitated. This facilitation is reflected in faster responses to targets in SP conditions than to targets at a different position (DP) than the cue. This facilitative cueing effect presumably reflects the capture of attention by the cue [11]. As explained, target perception requires that attention is shifted towards target locations [3,6]. Since the cue captures attention towards its location, in SP conditions attention is already at the target position. In contrast, in DP conditions attention has to be directed away from the cue to the target position, which can only be performed after target onset. This capture effect (*i.e.*, an SP-DP performance difference) is found with single abrupt-onset cues that are not predictive for the most likely target position [9,10] and it turns into Inhibition Of Return (IOR)—that is, slower responses for SP than DP targets—with SOAs beyond 300 ms [12,13,14]. IOR could be due to a sequence of capture by the cue, subsequent deallocation of attention away from the cue during the longer SOA, and a reluctance to reorient to the cued position if the target is finally presented there [13,15]. IOR might also reflect motor inhibition [14] or result from perceptual adaptation [12]. 

Whatever the exact origin of IOR, in line with subliminal attention, both the facilitative cueing effect with the short SOA and IOR with longer SOAs have also been found with subliminal abrupt-onset cues that were not seen by the participants (*i.e.*, could not be reported with better than chance accuracy) [7,8,15,16]. For example, presenting one disk as a single abrupt-onset cue on the left or on the right of a computer screen with a head start of 16 ms before two additional disks (at screen center and on the opposite screen side), this abrupt-onset cue can barely be seen: Due to the short onset difference, the participants were unable to decide whether a cue was presented on the left or on the right during a cue-discrimination task at the end of the experiment [15]. These subliminal cues captured attention [15,17]: Target detection was facilitated with a short cue-target SOA of 16 ms in SP conditions compared to DP conditions. In addition, with a longer SOA of 1,016 ms the subliminal cue led to IOR [15,17].

Exactly how the brain’s visual system brings about subliminal vision in general and subliminal attention in particular, however, is currently debated. One party of researchers thinks that at least some forms of unconscious vision reflect processing along the visual system’s parvocellular projection, leading from the retina to the cortex, via the Lateral Geniculate Nucleus (LGN) of the thalamus [18], and many forms of unconscious visual processing are probably of cortical origin [19,20]. 

However, another party of researchers recently proposed that subliminal attention at least partly reflects contrast-elicited attentional capture mediated by midbrain structures, namely neurons in the Superior Colliculi (SC) [15,17]. Functionally, the SC has a prominent role in the elicitation of saccadic eye movements and it contains cells sensitive to visual contrast and motion [21,22,23]. Anatomically, the SC is part of the magnocellular projection leading from the retina to the human cortex (via the nucleus pulvinaris of the thalamus), and it receives its input mainly from luminance-sensitive retinal ganglion cells [24,25]. As a consequence of these characteristics, the SC is very sensitive to the visual *contrast changes* elicited by luminance transients and abrupt visual onsets. 

In line with a collicular origin of subliminal attention, the unconscious cueing effect occurs when contrast increases (e.g., produced by sudden onsets) and independently of contrast polarities (*i.e.*, for both, lighter and darker items) [26]: When presenting subliminal light or dark rings (with the same Weber contrast but different signs) as abrupt-onset cues, similar facilitative cueing effects for both contrast polarities but no IOR were found [27]. Also, in general agreement with the hypothesis that the SC could be responsible for subliminal capture, the SC seems to (partly) account for “blindsight”—that is, the spared subliminal visual capabilities concerning input from visual field areas of scotoma after damage to primary visual cortex [28,29], but see [30]—and the SC has an active modulating role in visual attention [31].

**Figure 1:**
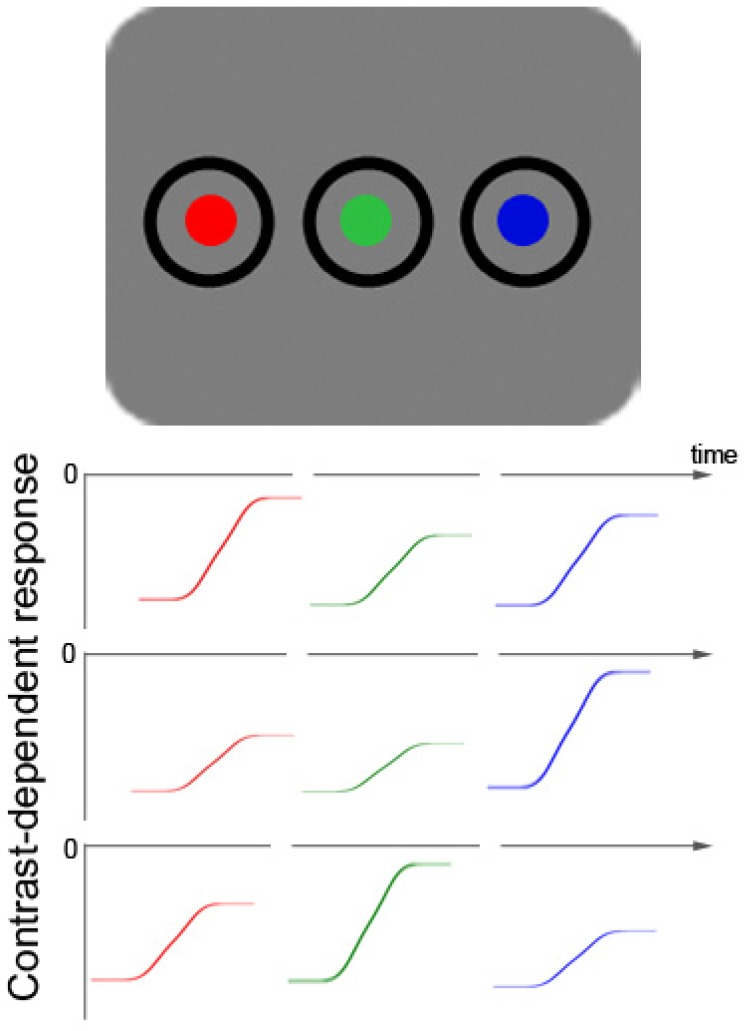
Schematic picture of a color-target display with two additional color distractors (top row). Below that, fictitious color-contrast dependent response functions for three different participants are plotted for all three different hues (rows 2–4). Colors with objectively the same luminance are able to produce different subjectively perceived contrasts in different observers due to varying sensitivity to particular hues. The figure demonstrates how three fictitious participants differ in their sensitivity and have their respective maximal contrast-dependent response in the range of red (row 2), blue (row 3), or green (row 4) colors. This figure illustrates the heterogeneity of the resulting contrast differences between participants, as well as the fact that the contrast differences are relatively small. The small size of the color contrast differences can be inferred from a comparison of the respective sizes of differences of: (1) background luminance responses (at 0) and the asymptotic maximum value of each color-elicited response; and (2) the background and black (this is the starting value of each color-elicited contrast-dependent response function). Figure 1 also depicts the way in which colors of targets and distractors were realized in the current Experiments 4 to 6: as changes from black to red, green, or blue.

Probably also in line with the SC hypothesis of subliminal attention are the differences between subliminal cueing by single abrupt-onset cues on the one hand [7,15,16] and by subliminal color cues on the other [32,33]. As explained, subliminal abrupt-onset cueing is found with abrupt-onset targets [7,15,16], and regardless of cue-target “color” (or luminance polarity) [27]. This is different with subliminal color cues during color search—that is, searches for a color-defined target: Subliminal cues with a color similar to the target capture attention but subliminal cues with a color different from the searched-for target fail to capture attention [32,33]. For example, if the target is red and presented together with either of three differently colored distractors (e.g., a green, a blue, and a gray distractor), it is difficult (if not impossible) for the participants to search for contrasts to find the target. The reason for this difficulty and the resulting cueing effect differences is probably simple. In comparison to a single abrupt-onset target that creates a single new contrast change, during color search each of the color stimuli (target and distractors) creates an (individually varying) different contrast change. This is true even if all stimuli are objectively equated for their luminance because individuals differ in their sensitivity for different colors [34,35] (see Figure 1). 

As a consequence of at least two simultaneous contrast changes during color search, the differences between the individual contrasts of the color-defined target on the one hand and the individual contrasts of the misleading color distractors on the other are mitigated (in comparison to a situation with a single abrupt-onset target). Even worse, the target could be of a lower contrast than at least some of the color distractors. Say, for example, that the target is blue. In this situation, if a participant is more sensitive to green than to blue colors (see Figure 1, 4th row), it would be difficult for this person to prioritize search for the target by searching for the target’s contrast. A low-contrast target among higher contrast-distractors could not be prioritized on the basis of the collicular contrast response because the collicular response is proportional to the strength of the contrast [26,36]. In this situation, the balance of attention-guiding channels in the brain’s network of biased competition [37,38] would therefore be tipped away from contrast as an attention-guiding principle and towards color. This is simply because the smaller contrast differences between relevant color target and irrelevant color disractors would be discriminated too late to dominate over the stronger and easier to discriminate color differences. In line with this, prior studies have shown that participants actually disregard small luminance differences during a color discrimination task [39]. In addition, because at least the superficial layers of SC are color-blind [40], the balance would thus also be tipped away from the SC and the magnocellular projection towards color-sensitive regions of the human visual system in the parvocellular projection (e.g., V2 [41]). In the following we refer to this possibility as the “SC hypothesis”, meaning here that contrast-driven subliminal capture based on the SC could also be shunted once color differences among relevant targets and irrelevant distractors are strong and contrast or luminance differences are weak.

### Overview of the Experiments

**Figure 2:**
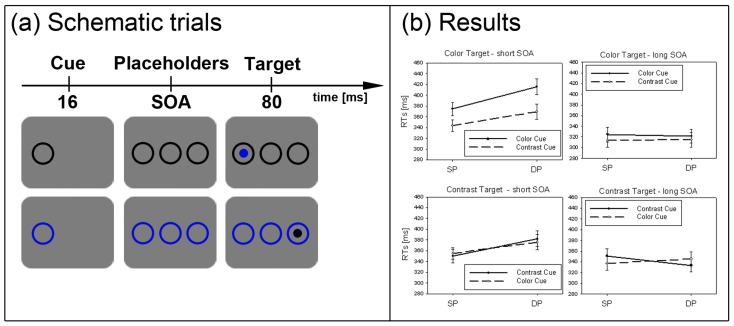
Experiment 1. (**a**) Depicted are schematic examples of trials. The top row shows a contrast cue and a blue target (same position (SP) condition). The bottom row depicts a color cue and a black target (different position (DP) condition). (**b**) Depicted are mean reaction times (RTs) and standard errors of the mean (error bars) of all participants, plotted separately for the short (left side) and long Stimulus Onset Asynchrony (SOA) (right side) and for color targets (top row) and contrast targets (bottom row). Results are shown for SP (left side of each panel) and DP conditions (right side of each panel). Results are plotted separately in each panel for cues of the same contrast or color (solid line) and different contrast or color cues (dashed line).

In the current study, we set out to test the SC hypothesis. First, we had to establish that under standard subliminal color cueing conditions with abrupt onset targets, it is possible to find a subliminal cueing effect. So far, it is unclear whether subliminal color cues capture attention when participants can search for a contrast-defined abrupt onset target. This possibility is tested in the present Experiment 1. For each trial, we presented a single subliminal color cue or contrast (black) cue (see also Figure 2). Importantly, the luminance of the color cue was objectively equated to the luminance of the gray background. Participants had to search for an abrupt-onset dark (*i.e.*, black) target or for a single color target. The black target can thus be located by its luminance or contrast change. Likewise, if it is true that the color target elicits an (individually varying) contrast, the color target could also be located by its contrast change. In this situation, we expected subliminal capture by the contrast cue and by the color cue. Due to the participants’ individual differences in their color sensitivities [34,35,42], the color cues should have led to a single luminance change. Also, because subliminal capture should not depend on contrast polarity [27], it should not have mattered whether the individual color sensitivities led to a positive or to a negative contrast polarity between color cue and background. Therefore, any contrast change elicited by the subliminal color cue was expected to capture attention when the relevant black target could be located by its contrast change (or its abrupt onset). (Of course, the black contrast cues which were darker than the background were expected to attract attention on the basis of their elicitation of a contrast change anyway. They were included as a safeguard to verify the sensitivity of our methods in the case that color cues failed to attract attention.) 

Second, the situation should dramatically change when the target-elicited luminance- or contrast-changes are smaller and therefore cannot be used as easily to locate the target. Since the attentional effect depending on the SC is assumed to be gradually dependent on contrast strength [36], cueing effects should be stronger for high- than low-contrast cues. This prediction was tested in Experiment 2. 

Third, another prediction by the SC hypothesis concerns the naso-temporal hemifield asymmetry of the retinal projection to the SC. Because the retino-tectal projection is much stronger for the temporal compared to the nasal aspect of the projection, on the basis of the SC hypothesis, with contrast cues and contrast-defined targets a stronger cueing effect after temporally than nasally presented cues was expected [43]. This hypothesis concerning the naso-temporal asymmetry of the subliminal cueing effect was tested in Experiment 3.

Experiment 4 tested whether abrupt-onset cues have the potential to capture attention during color search. According to the SC hypothesis, this should not be the case. The test was achieved by two measures, (A) and (B) (see also Figure 1). (A) We used three black disks, only one of which turned into the color-defined target (e.g., a red stimulus). Importantly, at target onset all disks simultaneously changed their colors. As a consequence, the color target was presented together with two color distractors of different colors. For example, if the target turned red, one distractor became blue, and the other green. In this way, the color-elicited contrast changes of all disks, including the target, were relatively small (in comparison to the contrast between the black disks and the background). (B) The relevant color target was unveiled by a color change at the same time as the two color changes in the additional placeholders. In this situation, for at least those participants that were less sensitive for the target colors than for either of the distractor colors, it would have been difficult to prioritize the localization of the target by searching for color-elicited contrasts. In Experiment 4, the subliminal SC contrast-capture effect was therefore expected to be shunted, and no subliminal cueing effect was expected even though a single subliminal black abrupt-onset cue of high cue-background contrast was used. We also ran a control experiment to test whether top-down singleton search provided a better account of our results (Experiment 5). This will be explained in more detail below. 

Finally, Experiment 6 was yet another control. We tested whether top-down contingent capture by color could account for the absence of the subliminal cueing effects under color-search conditions [44]. To that end, we used color-change cues instead of abrupt-onset cues, when the participants searched for a color-change target. If top-down contingent capture by color provided a better account of the lacking cueing effects of subliminal onset cues under color-search conditions, a cueing effect was to be expected in control Experiment 6 at least for the cues that changed into the same color as the target. This prediction holds true for clearly visible stimuli [44]. However, if the SC hypothesis of subliminal capture by abrupt-onset cues holds true, no cueing effect was to be expected in Experiment 6, too, because the targets (and cues) were defined as color changes and the SC is only sensitive to contrasts of abrupt-onset cues but color-blind.

## 2. Experimental Section

### 2.1. Experiment 1

In Experiment 1, we investigated whether subliminal color cues and subliminal (black) contrast cues both captured attention. Half of the participants searched for (black) contrast targets and half of the participants searched for color targets. In all conditions, we presented subliminal cues before the targets. We expected subliminal cueing effects. According to the hypothesis that subliminal attention is brought about by the SC [15,17], subliminal cueing effects should have been found because contrasts account for the SC-based subliminal cueing effect [26,27], and color cues created (individually varying) contrasts relative to the backgrounds even when equated for their objective luminance with the background [35,42]. 

#### 2.1.1. Participants

Twenty-four participants (18 female, mean age: 22.8 years) took part. Here and in subsequent experiments, participants were mostly students and participated on a voluntary basis in return for course credit. Participants in all our experiments had normal or corrected-to normal vision, as well as normal color-vision as assessed by Ishihara color plates. The procedure was explained prior to data acquisition, and informed consent was obtained from each participant.

#### 2.1.2. Stimuli and Procedure

Stimuli were presented on a 19-inch TFT screen at a viewing distance of 64 cm. Head position and viewing distance were supported by a chin rest with a forehead strip.

For our tests, we adapted the procedures of Mulckhuyse *et al.* [15]. Figure 2a shows example trials for Experiment 1. A trial started with a fixation cross, followed by a blank screen for 200 ms. Then the cue (a ring of the size of 3.0 × 3.0° and a stroke width of 0.25°, at 6.7° to the left or right of the centre) was presented for 16 ms, before two other placeholders (two additional rings) appeared at screen centre and on the opposite side. The target (a disk of 1.9 × 1.9°, equally likely in the left or right ring) was shown at the same position (SP) as the cue or at a different position (DP) than the cue. The target was either presented along with the other placeholders (short SOA of 16 ms) or with a delay of 1 s after the placeholders (long SOA of 1016 ms). The target (as well as the cue and placeholder rings) remained on the screen for 80 ms. 

Participants had two blocked tasks. In the first task (the subliminal cueing task), they had to report whether a target was presented by pressing the space bar of the computer keyboard. The target was presented in 80% of the trials. The participants had to refrain from pressing the bar when no target was shown. This was the case in the remaining 20% catch trials. In a second blocked task, cue visibility was assessed with the same participants. Here, the participants had to indicate at which position the cue was presented. They pressed one key on the left if the cue was on the left, and they pressed one key on the right if the cue was on the right.

In Experiment 1 (see Figure 2a), participants searched for a single abrupt-onset target, half of them for a black target (l = 23 cd/m^2^) and the other half for a predefined color target. Here, participants were informed by a written instruction on the screen to search for either a red (CLab color coordinates: 72.5/97.9/96.5), green (CLab: 72.5/−84.7/53.7), or blue (CLab: 72.5/22.2/−122.7) target. Target color varied between (and was balanced across) participants. Black and color targets were presented against a gray background (l = 72.5 cd/m^2^). With black targets, we used cues that were either black (same contrast: SC condition) or in one of the colors blue, red, or green (different contrast: DC condition). The color of the cue varied between (and was balanced across) participants. With color targets, we used cues with the same color (in the SC condition) or black cues (in the DC condition). 

Steps of the variables *cue position* (SP *vs.* DP), *cue contrast*/*color* (same *vs.* different), and *SOA* (short *vs.* long) were orthogonally combined and each combination was equally likely. The resulting conditions were realized in a random sequence of trials. Altogether participants had to work through 240 trials in the unconscious cueing task. The cue report task was the same except for its length (and the task) and conducted in four blocks consisting of 20 trials each (80 trials in total), directly after the unconscious cueing task. 

#### 2.1.3. Results

Trials with incorrect responses (*i.e.*, misses in target present trials and false alarms in target absent trials; 1.9%) as well as outliers (reaction times [RTs] deviating more than two standard deviations from the mean, further 2.7%) were excluded.

#### 2.1.4. Subliminal Cueing Task

Results are depicted in Figure 2b. A repeated measurements ANOVA with the within-participant variables *cue position* (SP *vs.* DP), *cue contrast*/*color* (same *vs.* different), and *SOA* (short *vs.* long), and with *target type* (contrast *vs.* color) as a between-participants variable on mean correct RTs was calculated.

The subliminal cues captured attention. This was reflected in a significant main effect of cue position, *F*(1, 22) = 38.7, *p* < 0.001, η_p_^2^ = 0.64, with lower RT in SP (RT = 344 ms) than DP (RT = 357 ms) conditions. There was also a main effect of cue contrast/color, *F*(1, 22) = 20.2, *p* < 0.001, η_p_^2^ = 0.48, with slower responses in same (RT = 357 ms) than different contrast/color conditions (RT = 344 ms). Finally, there was a significant main effect of SOA, *F*(1, 22) = 30.7, *p* < 0.001, η_p_^2^ = 0.58. In the long SOA, participants benefited from a longer preparation time (RT = 330 ms) as compared to the short SOA (RT = 371 ms).

Importantly, a significant three-way-interaction between the variables SOA, cue position, and cue contrast/color, *F*(1, 22) = 13.8, *p* < 0.01, η_p_^2^ = 0.39, and a significant two-way interaction for cue position and SOA, *F*(1, 22) = 74.3, *p* < 0.001, η_p_^2^ = 0.77, pointed to different strengths of the cueing effects depending on SOA and cue contrast/color. In addition, three significant interactions with the between-participants variable target type (black *vs.* color) were found: (1) with cue contrast/color, and SOA, *F*(1, 22) = 13.3, *p* < 0.01, η_p_^2^ = 0.38, (2) with cue contrast/color, *F*(1, 22) = 17.8, *p* < 0.001, η_p_^2^ = 0.48, and (3) with SOA, *F*(1, 22) = 5.2, *p* < 0.05, η_p_^2^ = 0.19. We therefore decided to run separate *post-hoc* Bonferroni-adjusted *t*-tests to compare SP to DP conditions (or test the significance of cueing effects) separately for each combination of the levels of the variables target type, cue contrast/color, and SOA. With the short SOAs, these *t*-tests revealed cueing effects (*i.e.*, lower RTs in SP than DP conditions) for both target types, contrast- and color-defined targets, and either sort of cue (same and different contrast/color) (all *p*s < 0.01). With the long SOA, the *post-hoc* tests only confirmed a significant IOR effect with black targets following a same contrast/color cue (*p* < 0.05). All other SP-DP differences in the long SOA fell short of significance.

#### 2.1.5. Visibility of Subliminal Cues and Its Correlation with the Cueing Effects

After the first block, participants were informed that a cue appeared one frame earlier than the two other placeholders in each trial. All participants reported that complete unawareness of the cue in the subliminal cueing task, indicating subjective invisibility of the cues, and hence unconscious processing [45]. Objective cue visibility was assessed with the same participants based on their performance in a separate block. Here, the task was to report at which of two possible positions the cue was shown (in a forced-choice task). The sensitivity measure *d’* was calculated for only those trials in which a target was presented. Correct reports of cues on the right counted as hits, and incorrect reports of cues on the right as false alarms, and *d’* was calculated as the difference between the *z-*transformed probabilities of the hits minus the *z-*transformed probabilities of the false alarms. *d’* will be zero in the case of chance performance (or invisibility of the cues) and can infinitely increase with an ever increasing discrimination performance. 

To assess objective cue discrimination, we calculated the sensitivity measure *d’* and the response criterion *c* for the target present trials. Mean *d’* (=1.9; range from −0.1 to 4.0) was significantly above chance-level (*p* < 0.001); mean *c* (=0.1; range from −0.5 to 1.3) did not significantly differ from zero (*p* = 0.37). 

**Figure A1:**
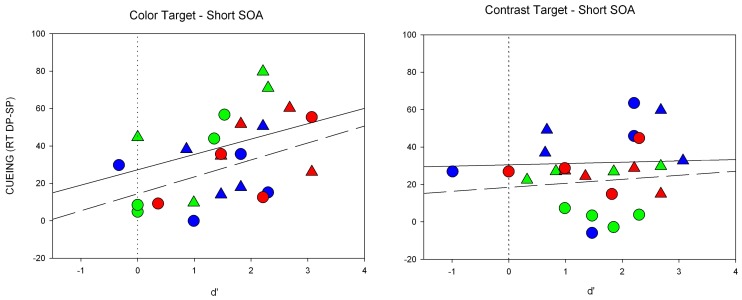
Individual scatter plots for Experiment 1. Shown are RT differences (*y*-axis) and the respective *d’*-value (*x*-axis) for each participant separately. Panels depict results of the short SOA (cueing effects, *i.e.*, mean RTs for DP minus RTs for SP condition). Values are plotted separately for same contrast/color conditions (triangles, solid regression line) and different color/contrast conditions (circles, dashed regression line). For color targets (panel on the left side), the symbols are plotted in the corresponding target color, for contrast targets (panel on the right side) the color of the symbols correspond to the cue color.

To assess whether the cueing effect obtained under zero discrimination conditions, we calculated regressions of the cueing effect (*i.e.*, RTs for DP-SP conditions) as a function of discrimination (*d’*) for the short SOA (see also Figure A1 in the Appendix). Cueing effects at zero discrimination will be reflected in a significant offset of the regression (for further details see [46]). Importantly, a significant intercept of the regression indicated that the RT cueing effect was significantly above 0 (*a* = 22.1, *p* < 0.01) when *d’* was equal to zero, whereas no significant correlation obtained between discrimination performance and cueing effect (beta = 0.30, *p* = 0.15). 

#### 2.1.6. Discussion

The major result of Experiment 1 is in agreement with the SC hypothesis of subliminal attention. Subliminal color cues captured attention, regardless of whether they were of the same color as the targets or not. This result corresponds to previous findings demonstrating a contrast-polarity independence of the subliminal cueing effect under very similar conditions [27]. The result thus also demonstrated that the lack of subliminal cueing effects in studies with color-defined targets [32,33] cannot be due to the relative weak contrast-response elicited by subliminal color cues. In fact, in the present experiment, we observed no significant differences in the cueing effects of the short SOA conditions between color cues and contrast cues, although the latter were maybe of a higher average cue-background contrast. For example, as all of our participants passed the Ishihara color test, our sample of participants did not contain any extreme cases, like red-green color blind persons for whom a red stimulus would have been of a black appearance, too. This finding of an independence of the subliminal cueing effect from contrast strength is not fully in line with the assumed dependence of SC-mediated attention effects on contrast strength [26,36]. To note, however, we actually did not measure individually perceived color contrasts and therefore do not know how strong the differences between color cues and black cues really were.

A second result of Experiment 1 that is equivocal with respect to our hypothesis that the SC and the magnocellular projection are responsible for subliminal cueing is the lack of IOR in most of the long SOAs. Only when participants searched for a black target (see Figure 2a, right panel), SC cues led to IOR. This lack of IOR is puzzling but it is not without precedence. For example, in previous studies, researchers also failed to find IOR under conditions very similar [27] or even identical [47] to studies in which IOR was reported [15,17]. We do not have an explanation for this difference between studies. However, we think that the subliminal cueing effect with short SOAs could simply be more robust than the subliminal IOR effect. This would explain why IOR effects after subliminal cues are also elusive in studies that used different experimental protocols [7,48] (although some instances of absent subliminal IOR effects could also be explained in yet another way [16]).

### 2.2. Experiment 2

In Experiment 2, we once again tested the dependence of attentional effects on contrast strength as predicted by the SC hypothesis. According to the hypothesis that the SC accounts for the subliminal cueing effect, cues of a higher contrast should result in stronger cueing effects compared to lower contrast cues [26,36]. For our tests, we varied the contrast polarity and strength of the cues independently of the searched-for target contrast.

#### 2.2.1. Participants

Eight naïve participants (all female, mean age: 20.5 years) took part in Experiment 2.

#### 2.2.2. Stimuli and Procedure

**Figure 3:**
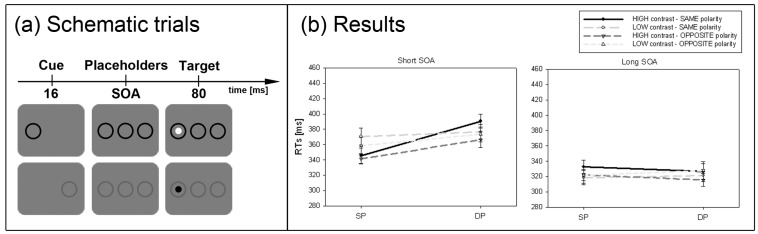
Experiment 2. (**a**) Depicted are examples of schematic trials. The top row depicts an opposite-polarity cue at the same position as the target in the high-contrast condition (black cue and white target). The bottom row shows a same-polarity cue in the low-contrast condition (DP condition). (**b**) Depicted are mean RTs and standard errors of the mean (error bars) of all participants for the short (left panel) and long SOA (right panel). Results are plotted for all conditions separately (see figure legend).

The general procedure was similar to Experiment 1, except that only contrast-defined targets were used (see Figure 3a). Half of the participants were instructed to search for black targets (l = 23 cd/m^2^), the other half for white targets (l = 122 cd/m^2^) against a gray background (l = 72.5 cd/m^2^). Both target types had the same Weber Contrast, but with different signs (*i.e.*, C_w_ = −0.7 for black targets and, C_w_ = +0.7 for white targets). Cues varied with respect to their contrast polarity and strength: high-contrast cues were equally likely of the same contrast as the target or the opposite contrast than the target, with luminance values as reported above for the targets. Additionally, we used low-contrast cues of both polarities, *i.e.*, lighter (l = 76.5 cd/m^2^) and darker (l = 68.5 cd/m^2^) than the background (C_w_ = ±0.055). Again, cues were equally likely presented at the same or the different position as the target. In 20% out of 320 trials in the subliminal cueing task no target was shown (catch trials). The cue report task was conducted in four blocks consisting of 20 trials each (80 trials in total), directly after the unconscious cueing task. 

#### 2.2.3. Results

Trials with incorrect responses (1.3%) and RT outliers (further 3.4%) were again excluded from further analysis. 

#### 2.2.4. Subliminal Cueing Task

Results are depicted in Figure 3b. A repeated measurements ANOVA with the factors *cue position* (SP *vs.* DP), *cue contrast polarity* (same *vs.* different), *cue contrast strength* (low *vs.* high contrast) and *SOA* (short *vs.* long) on mean correct RTs led to the following results. We found a significant interaction between cue position and SOA, *F*(1, 7) = 19.0, *p* < 0.001, η_p_^2^ = 0.73. *Post-hoc* Bonferroni adjusted *t*-tests revealed a significant cueing effect (*i.e.*, faster responses to SP than DP cued targets) for the short SOA (SP: RT = 354 ms, DP: RT = 377 ms, *p* < 0.01), whereas no effect was found for the long SOA (SP: RT = 324 ms, DP: RT = 323 ms, *p* = 0.79). The significant interaction between cue position, contrast strength and SOA, *F*(1, 7) = 11.6, *p* < 0.05, η_p_^2^ = 0.62, and *post-hoc* Bonferroni adjusted *t*-tests revealed that the cueing effect was stronger for short-SOA high-contrast cues (SP: RT = 343 ms, DP: RT = 379 ms, *p* < 0.01) than for short-SOA low-contrast cues (SP: RT = 365 ms, DP: RT = 375 ms, *p* < 0.05). No significant effects were found in the long SOA for both, high and low-contrast cues (both *p*s > 0.14). We also found a significant interaction between cue position, cue polarity and contrast strength, *F*(1, 7) = 10.3, *p* < 0.05, η_p_^2^ = 0.60. Follow-up Bonferroni adjusted *t*-tests revealed that for high-contrast cues of the same polarity responses to SP cues (RT = 339 ms) were generally faster than to DP cues (RT = 359 ms; *p* < 0.001), whereas all other conditions failed to reach significant effects (all *p*s > 0.06). Furthermore, we found a significant main effect for cue position, *F*(1, 7) = 17.5, *p* < 0.001, η_p_^2^ = 0.71, reflecting that responses in SP conditions (RT = 339 ms) were generally faster than in DP conditions (RT = 350 ms). The main effect for contrast polarity, *F*(1, 7) = 7.6, *p* < 0.05, η_p_^2^ = 0.52, indicated overall faster responses to the opposite- (RT = 341 ms) than to same-polarity cues (RT = 348 ms). Finally, the main effect for SOA, *F*(1, 7) = 112.3, *p* < 0.001, η_p_^2^ = 0.94, confirmed that participants responded faster in the long SOA (RT = 323 ms) than the short SOA (RT = 365 ms).

#### 2.2.5. Visibility of Subliminal Cues and Its Correlation with the Cueing Effects

Again, all participants reported having been subjectively unaware of the cues after the first block. To assess objective cue discrimination, we calculated the sensitivity measure *d’* and the response criterion *c* for the target present trials. Mean *d’* (=0.9; range from −0.4 to 2.0) was significantly above chance-level (*p* < 0.05); mean *c* (=−0.2; range from −0.5 to 0.3) did not significantly differ from zero (*p* = 0.16).

**Figure A2:**
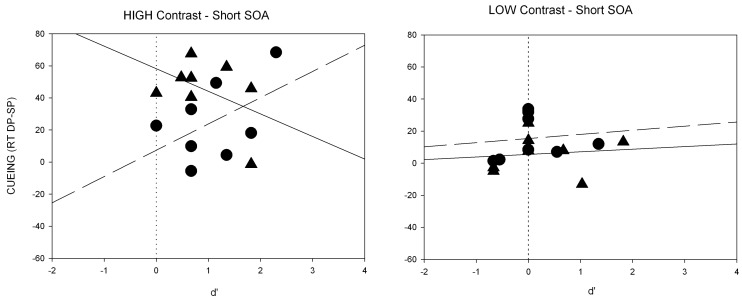
Individual scatter plots of the short SOA for Experiment 2. Shown are RT differences (cueing effects, *i.e.*, mean RTs for DP minus RTs for SP condition on the *y*-axis) and the respective *d’*-value (on the *x*-axis) for each participant separately. Panels depict results for high-contrast (left side) and low-contrast cues (right side), separately. In each panel values are displayed for cues with the same polarity as the target (triangles; solid regression line) and with opposite polarity (circles; dashed regression line).

We calculated regressions of the RT differences between the SP and DP conditions as a function of discrimination (*d’*) for the short SOA (see also Figure A2 of the Appendix). Again, a significant intercept of the regression was found, indicating that the RT cueing effect (RTs for DP-SP trials) was significantly above zero (*a* = 15.9, *p* < 0.05) when *d’* was equal to zero, whereas no significant correlation between discrimination performance and cueing effect (beta = 0.50, *p* = 0.21) was found. 

#### 2.2.6. Discussion

The results are in line with the SC-hypothesis of subliminal attention: for the short cue-target interval we found stronger cueing effects for high-contrast than low-contrast cues. Furthermore, we found no significant effect of contrast polarity on cueing. This fact additionally supports the bottom-up hypothesis of attention, which assumes that attention is captured by a high-contrast abrupt-onset cue regardless of the sign of its contrast [49,50].

### 2.3. Experiment 3

Another prediction of the SC hypothesis is a naso-temporal asymmetry of the cueing effect [43]. Attentional effects under monocular viewing conditions should be stronger for cues presented in the temporal compared to the nasal hemifield [51]. This behavioral asymmetry is predicted on the basis of the asymmetry of the retinotectal pathway: The SC receives its input primarily from the temporal visual field [52]. Furthermore, it has been shown, that the SC shows a larger response to stimuli presented in the temporal than nasal visual field, whereas other structures failed to show this temporal hemifield advantage (e.g., LGN or the visual cortex) [53]. This asymmetry was tested under monocular viewing conditions.

#### 2.3.1. Participants

Eight naïve participants (7 female, mean age: 24.0 years) took part in Experiment 3.

#### 2.3.2. Stimuli and Procedure

**Figure 4:**
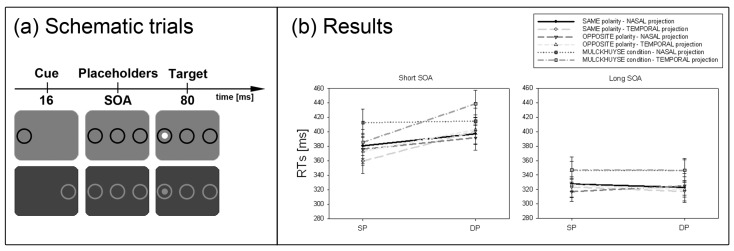
Experiment 3. (**a**) Depicted are schematic examples of trials. The top row depicts an opposite-polarity cue at the same position as the target (black cue and white target). The bottom row shows a DP cue in the Mulckhuyse condition (where cue and target always shared the same contrast polarity). Note, that the depicted luminance of the items is just for illustration purposes and does not equal the original luminance. Furthermore, the cues, targets, and outer rings were presented at an increased distance to the centre compared to all other experiments. (**b**) Depicted are the mean RTs and standard errors of the mean (error bars) of all participants for the short (left panel) and long SOA (right panel). Results are plotted for all conditions separately (see figure legend).

The general procedure was similar to Experiment 2 (see Figure 4a). Crucially, the distance of the two outer rings as well as that of the targets to the centre was increased (*i.e.*, 10° to the left or right of the centre). This was necessary to adjust our procedures to the known retinotopy of the naso-temporal retino-tectal projection asymmetry. Again, half of the participants were instructed to search for black targets (l = 23 cd/m^2^), the other half for white targets (l = 122 cd/m^2^) against a gray background (l = 72.5 cd/m^2^). Additionally, all participants searched for targets under the exact same luminance conditions as in the study of Mulckhuyse *et al.* [15] in a separate block. This condition will henceforth be referred to as *Mulckhuyse condition.* In this condition, cues always shared the same contrast polarity and strength as the target (l = 12.7 cd/m^2^) against a dark background (l = 4.6 cd/m^2^). Therefore, in Experiment 3, the variable *cue condition* was realized in three steps: same polarity, opposite polarity, and Mulckhuyse condition. Again, cues were presented equally likely at the same or a different position as the target. In 20% out of 480 trials in the subliminal cueing task no target was shown (catch trials). The cue report task was conducted in four blocks consisting of 40 trials each (160 trials in total), directly after the unconscious cueing task. Monocular viewing conditions were established using an eye patch. The placement of the patch over the left or right eye was fully balanced over participants. Furthermore, the position of the patch was switched after the first half of the trials of each condition.

#### 2.3.3. Results

Trials with incorrect responses (2.7%) and RT outliers (further 5.2%) were again excluded from further analysis. 

#### 2.3.4. Subliminal Cueing Task

Results are depicted in Figure 4b. A repeated measurements ANOVA with the factors *cue position* (SP *vs.* DP), *cue condition* (same polarity *vs.* opposite polarity *vs.* Mulckhuyse condition), *hemifield projection* (nasal *vs.* temporal) and *SOA* (short *vs.* long) on mean correct RTs led to the following results. We found a significant interaction between cue position and SOA, *F*(1, 7) = 29.9, *p* < 0.01, η_p_^2^ = 0.81. *Post-hoc* Bonferroni adjusted *t*-tests confirmed a cueing effect in the short SOA (SP: RT = 381 ms, DP: RT = 408 ms, *p* < 0.01) and no significant effect in the long SOA (SP: RT = 331 ms, DP: RT = 330 ms, *p* = 0.61). As indicated by the significant interaction between cue position, hemifield projection and SOA, *F*(1, 7) = 11.1, *p* < 0.05, η_p_^2^ = 0.61, and confirmed by *post-hoc* Bonferroni adjusted *t*-tests, the above reported cueing effect was only evident when the cue was projected to the temporal hemifield (SP: RT = 373 ms, DP: RT = 414 ms, *p* < 0.01), but not for the nasal projection (SP: RT = 390 ms, DP: RT = 401 ms, *p* = 0.26). Furthermore, we found a significant main effect for cue position, *F*(1, 7) = 21.0, *p* < 0.01, η_p_^2^ = 0.75, reflecting that responses in SP conditions (RT = 356 ms) were generally faster than in DP conditions (RT = 369 ms). The main effect for cue condition, *F*(2, 14) = 13.4, *p* < 0.01, η_p_^2^ = 0.66, indicated overall slower responses in the Mulckhuyse condition (only same polarity: RT = 380 ms) compared to the other conditions (same polarity: RT = 354 ms, opposite polarity: RT = 354 ms). Finally, the main effect for SOA *F*(1, 7) = 79.0, *p* < 0.001, η_p_^2^ = 0.92, confirmed that participants responded faster in the long SOA (RT = 330 ms) than the short SOA (RT = 395 ms).

#### 2.3.5. Visibility of Subliminal Cues and Its Correlation with the Cueing Effects

All participants reported that they have been completely unaware of the cue (*i.e.*, the one ring starting one frame earlier) in the first block. To assess objective cue discrimination, we calculated the sensitivity measure *d’* and the response criterion *c* for the target present trials. Mean *d’* (=0.5; range from 0.2 to 1.2) was significantly above chance-level (*p* < 0.01); mean *c* (=0.1; range from −0.3 to 0.3) did not significantly differ from zero (*p* = 0.38).

**Figure A3:**
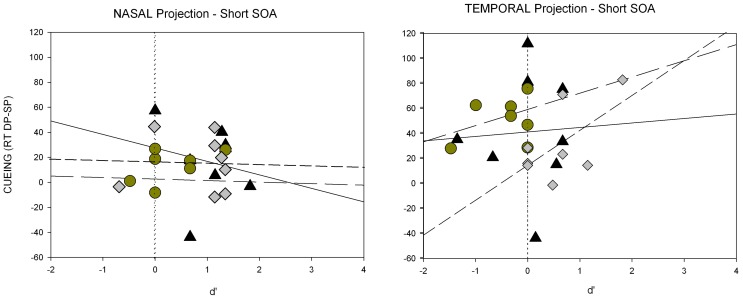
Individual scatter plots of the short SOA for Experiment 3. Shown are RT differences (cueing effects, *i.e.*, mean RTs for DP minus RTs for SP condition on the *y*-axis) and the respective *d’*-value (on the *x*-axis) for each participant separately. Panels depict results separately for nasal (left side) and temporal hemifield projection (right side). Within each panel, values for all three cue conditions are shown: same polarity cues (triangles; solid regression line), opposite polarity cues (diamonds; short dashed regression line) and Mulckhuyse condition (circles; long dashed regression line).

We calculated regressions of the RT differences between the SP and DP conditions as a function of discrimination (*d’*) for the short SOA (see also Figure A3 of the Appendix). Again, a significant intercept of the regression was found, indicating that the RT cueing effect (RTs for DP-SP trials) was significantly above 0 (*a* = 35.4, *p* < 0.01) when *d’* was equal to zero, whereas no significant correlation obtained between discrimination performance and cueing effect (beta = −0.54, *p* = 0.17). 

#### 2.3.6. Discussion

In line with the SC hypothesis, cueing effects in the short SOA were found only for cues presented to the temporal hemifield in all our conditions. This finding further supported the assumption that the SC mediates the subliminal attentional effects.

### 2.4. Experiment 4

**Figure 5:**
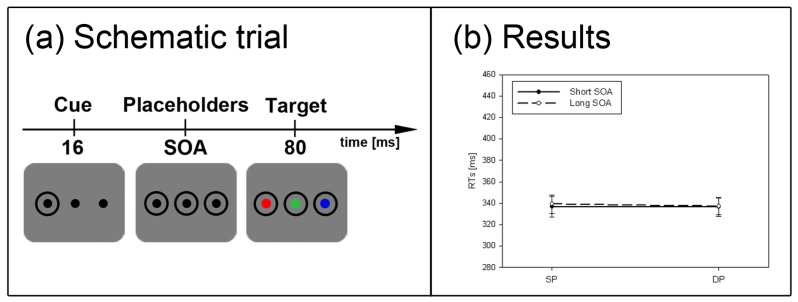
Experiment 4. (**a**) Depicted is a schematic example trial. For participants searching for a red target, the depicted trial is an example of the SP condition, whereas for participants searching for a blue target, the same trial depicts a DP condition. (**b**) Depicted are the mean RTs and standard errors of the mean (error bars) of all participants for the short (solid line) and long SOA (dashed line).

Having established subliminal attention with contrast cues and color cues under conditions in which targets can be located by their contrasts in the present Experiments 1 to 3, we turned to our test of the SC hypothesis. In Experiment 4, we used color-defined targets (red, green, or blue; between participants; for the respective CLab color coordinates see Experiment 1). Each color target was accompanied by two different color distractors. For example, if the color target was red, one distractor was green and one blue. The luminance of the color target and the color distractors was objectively the same as that of the background (all ls = 72.5 cd/m^2^). However, because of the participants’ individually varying sensitivity for different colors, all color stimuli had varying perceived contrasts—although in general rather low subjective contrasts. In this situation, the contrast responses that are elicited by the color distractors will functionally “mask” the contrast response elicited by the color target, (see Figure 1). In addition, targets and distractors were characterized as contrast reductions: At the beginning of each trial, three black disks or three white disks of high contrast were presented against the gray background. Then one disk turned into the color target and the others turned into the color distractors. Importantly, all these color changes came along with luminance increments of the black disks towards the background luminance (see Figure 5a) or with luminance decrements of the white disks towards the background luminance. Thus, all these changes were contrast reductions.

Therefore, in Experiment 4, color-defined targets could not be as easily and reliably located by their contrasts. This in turn should have increased the influence of the color channels, if only because of a higher probability that color discrimination between target and distractors dominated over the more difficult and, thus, slower contrast discrimination. As a consequence of the more efficient color discrimination than contrast discrimination, the attentional influence of the parvocellular projection as the major color channel conveying the visual attentional effect should have increased, whereas the attentional influence of the magnocellular pathway and the SC as a major contrast channel should have been shunted. In other words, we expected to find no subliminal cueing effect by even a strong contrast cue. This hypothesis was tested with one single black abrupt-onset cue per trial. This cue should not have captured attention in Experiment 4, despite its capturing attention under the single-target conditions of Experiment 1.

#### 2.4.1. Participants

Twenty-four naïve participants (19 female, mean age: 23.1 years) took part in Experiment 4.

#### 2.4.2. Stimuli and Procedure

The general procedure was similar to Experiment 1. However, participants had to search for a predefined target color and responded to this color target. Also in contrast to Experiment 1, three additional disks were shown on the screen throughout each trial. For half of the participants, these disks (and the rings) were all black at the start of the trial. For the other half of the participants these disks (and the rings) were all white. After 16 ms (short SOA) or 1016 ms (long SOA) the target was presented—that is, one of the disks turned into a red, green, or blue color target. (Which of these color changes was the color target was balanced across participants.) Along with the color target, the two other disks also changed (from black or white) into the remaining two different distractor colors (see Figure 5a). Again, 20% out of 240 trials in the subliminal cueing task were catch trials without a color target.

#### 2.4.3. Results

Trials with incorrect responses (1.3%) and RT outliers (further 1.4%) were again excluded from further analysis. 

#### 2.4.4. Subliminal Cueing Task

Results are depicted in Figure 5b. A repeated measurements ANOVA with the factors *cue position* (SP *vs.* DP), and *SOA* (short *vs.* long) on mean correct RTs was calculated. We found no significant results. Furthermore, the current results were compared to a subgroup of Experiment 1 in which participants responded to a color-defined target that was cued by a black abrupt-onset cue (*N* = 12). A repeated measurements ANOVA with the factors *cue position* (SP *vs.* DP), and *SOA* (short *vs.* long), and the between-participants factor *experiment* (1 *vs.* 4) resulted in a significant three-way interaction between all variables, *F*(1, 34) = 10.5, *p* < 0.01, η_p_^2^ = 0.24. *Post-hoc* Bonferroni-adjusted *t*-tests confirmed a significant cueing effect only for Experiment 1’s short SOA (*p* < 0.001) but not for Experiment 4’s short SOA (*p* = 0.92), and no significant results in the long SOA of either experiment (both *p*s > 0.50).

#### 2.4.5. Visibility of Subliminal Cues and Its Correlation with the Cueing Effects

Participants in Experiment 4 again reported to have been subjectively unaware of the cues in the first block. As an objective measure of cue discrimination we calculated the sensitivity measure *d’* and the response criterion *c* for the target present trials. Mean *d’* (=1.8; range from 0.1 to 3.7) was significantly above chance-level (*p* < 0.001); mean *c* (=−0.1; range from −0.8 to 1.0) did not significantly differ from zero (*p* = 0.19).

**Figure A4:**
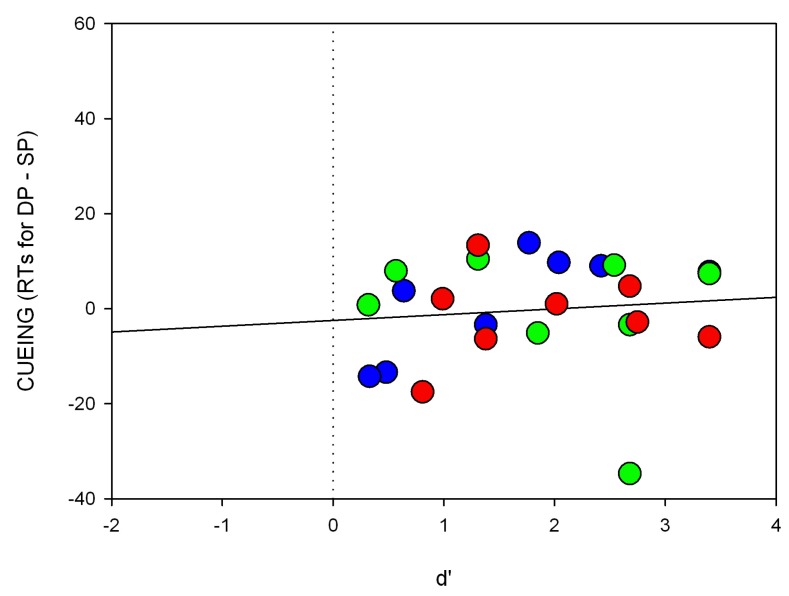
Individual scatter plots of the short SOA for Experiment 4. Shown are RT differences (cueing effects, *y*-axis) and the respective *d’*-value (*x*-axis) for each participant separately. The colors of the circles correspond to the respective searched-for target color.

As a sanity check of our invisibility measure, we also calculated regressions of the RT differences between the SP and DP conditions as a function of discrimination (*d’*) for the short SOA (cueing effect, *i.e.*, RTs for DP-SP trials; see also Figure A4 of the Appendix). We found that the intercept was not significantly above zero (*a* = −2.5, *p* = 0.62) and no significant correlation between discrimination and RT differences (beta = 0.11, *p* = 0.62).

#### 2.4.6. Discussion

The absence of a cueing effect in Experiment 4 is in line with our SC hypothesis. Experiment 4 showed that when target localization by color discrimination is required or easier than by contrast discrimination, the parvocellular color channels’ influence on attention increased and that of the magnocellular contrast channels decreased in a network of biased competition. As a consequence, abrupt-onset cues with a strong contrast and a color different from the targets failed to capture attention. This observation is in line with previous studies of subliminal cueing [32,33]. This finding is also in agreement with studies showing that abrupt onsets do not invariably capture attention in all instances [44,54,55]. Finally, the finding perfectly suits with the assumption that contrast-elicited capture by subliminal cues relies on the SC. Because at least the superficial layers of the SC are color-blind, the color discrimination could not be brought about by the SC and the SC pathway was therefore shunted.

### 2.5. Experiment 5

Experiment 5 was a control experiment to rule out that singleton search was responsible for the different results of our Experiments 1 and 4. In Experiment 1, the cue was a singleton—that is, the cue stood out by a unique feature (here: its abrupt onset). Therefore, the participants could have searched for the targets by singleton search. Suppose that the participants have set up attentional control settings in a top-down way to search for singletons [56]. If this happened in Experiment 1, the cue could have captured attention in a top-down contingent fashion, because of its fit to the attentional control settings [56]. In contrast, in Experiment 4, the cue was again a singleton but the target was a non-singleton. That is, the target neither stood out by its abrupt onset, nor by its color or any other of its features. Therefore the participants could not have searched for singletons to find the target. Thus, the singleton cue could also not have matched an attentional control setting to search for singleton targets. As a consequence of this difference between Experiments 1 and 4, the subliminal cueing effect in Experiment 1 could have reflected top-down contingent attentional capture by subliminal singletons and the lack of the effect in Experiment 4 could have reflected the absence of top-down control settings to search for singletons. 

Experiment 5 was a control for this possibility. Experiment 5 was the same as Experiment 4 but each target was a color singleton. For example, if one disk turned into a green target the two other disks both turned into blue distractors. In Experiment 5, the target therefore stood out by its color, and participants were able to search for target singletons. If top-down contingent capture for singletons accounted for subliminal attention, the results of Experiment 5 should have resembled those found in Experiment 1: A subliminal cueing effect of the abrupt-onset cue was expected. However, if the SC hypothesis accounted for our results, the cueing effects were expected to resemble the results of Experiment 4: No subliminal cueing effect was expected because (as in Experiment 4) only color differences would have been fit to locate the targets, whereas contrast differences would not have been fit to locate the targets.

#### 2.5.1. Participants

Twenty-four naïve participants (12 female, mean age: 24.1 years) took part in Experiment 5. 

#### 2.5.2. Stimuli and Procedure

**Figure 6:**
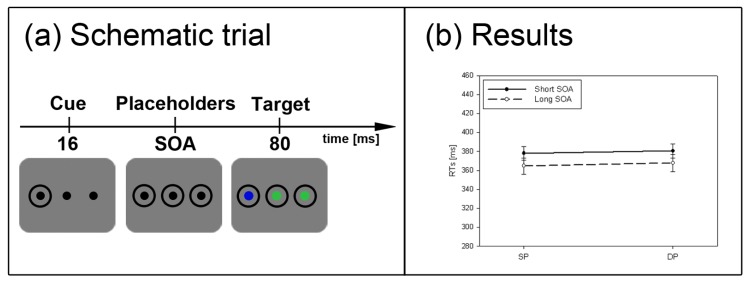
Experiment 5. (**a**) Depicted is a schematic example trial (blue target, SP condition). (**b**) Depicted are the mean RTs and standard errors of the mean (error bars) of all participants, plotted separately for the short (solid line) and long SOA (dashed line).

The procedure was identical to the one used in Experiment 4, except for one change: When the target disk (left or right) changed to the predefined target color, the disk on the other side of the screen as well as the centre disk both changed to the same distractor color (see Figure 6a). Therefore, participants could have searched for a singleton to find the target.

#### 2.5.3. Results

Trials with incorrect responses (3.3%) or outlying reaction times (further 3.1%) were excluded from further analysis. 

#### 2.5.4. Subliminal Cueing Task

Results are depicted in Figure 6b. A repeated measurements ANOVA with the variables *cue position* (SP *vs.* DP), and *SOA* (short *vs.* long) on correct mean RTs was calculated. It revealed a significant main effect for SOA, *F*(1, 23) = 11.4, *p* < 0.01, η_p_^2^ = 0.33, with generally shorter RTs in the long (mean RT = 366 ms) compared to the short SOA (mean RT = 379 ms). All other differences and effects fell short of significance. Again, the current results were compared to a subgroup of Experiment 1 in which participants responded to a color-defined target that was cued by a black abrupt-onset cue (*N* = 12). A repeated measurements ANOVA with the factors *cue position* (SP *vs.* DP), and *SOA* (short *vs.* long), and the between-participants factor *experiment* (1 *vs.* 5) resulted in a significant three-way interaction between all variables, *F*(1, 34) = 10.5, *p* < 0.01, η_p_^2^ = 0.24. *Post-hoc* Bonferroni-adjusted *t*-tests confirmed a significant cueing effect only for Experiment 1’s short SOA (*p* < 0.001) but not for Experiment 5’s short SOA (*p* = 0.47), and no significant results in the long SOA of both experiments (both *p*s > 0.22).

#### 2.5.5. Visibility of Subliminal Cues and Its Correlation with the Cueing Effects

Subjectively, participants were again unaware of the cues after the first block. To assess objective cue discrimination, we calculated the sensitivity measure *d’* and the response criterion *c* for the target present trials. Mean *d’* (=1.7; range from −1.4 to 4.3) was significantly above chance-level (*p* < 0.001); mean *c* (=−0.1; range from −0.7 to 0.5) did not significantly differ from zero (*p* = 0.08).

**Figure A5:**
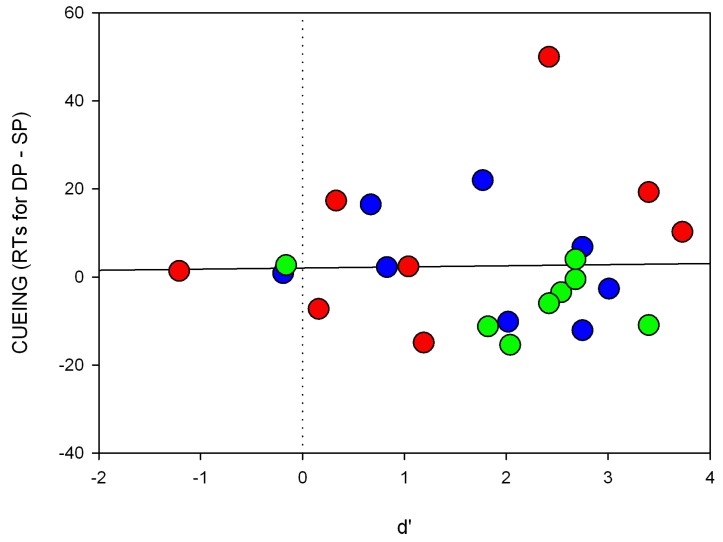
Individual scatter plots for Experiment 5. Shown are RT differences (cueing, *y*-axis) and the respective *d’*-value (*x*-axis) of the short SOA for each participant separately. The colors of the circles correspond to the respective searched-for target color.

We also again calculated regressions of the RT differences between the SP and DP conditions as a function of discrimination (*d’*) for the short SOA (cueing effect, *i.e.*, RTs for DP-SP trials; see also Figure A5 of the Appendix). We found that the intercept was not significantly different from zero (*a* = 2.0, *p* = 0.70) and no significant correlation between discrimination and RT differences (beta = 0.02, *p* = 0.91). 

#### 2.5.6. Discussion

When searching for a color singleton, abrupt-onset cues failed to capture exogenous attention. This is in line with prior findings with clearly visible cues [44]. The finding also supports our hypothesis that the SC is shunted if the color of the target is better suited to locate the target than the contrast of the target. 

### 2.6. Experiment 6

Top-down contingent capture for color rather than for onsets [44] might also better explain why an abrupt onset failed to capture attention when participants were actually searching for a color-change target (in Experiments 4 and 5). To test this hypothesis in Experiment 6, both, cues and targets, were realized as color-changes and the colors of cues and searched-for targets were identical in half of the trials and different in the other half of the trials. If top-down contingent capture by color provides a better explanation, a subliminal cue with a color similar to the searched-for target color should capture attention but a subliminal cue with a color different from the searched-for target color should fail to capture attention. However, if the subliminal cueing effect in Experiments 1 to 3 depended on the cue’s abrupt onset effect via the SC and if the color search necessitated a shunting of the SC, then no cueing effect was to be expected in Experiment 6, too.

#### 2.6.1. Participants

Twenty-four naïve participants (20 female, mean age: 28.0 years) took part in Experiment 6.

#### 2.6.2. Stimuli and Procedure

**Figure 7:**
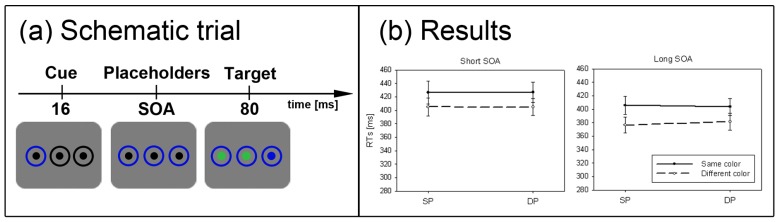
Experiment 6. (**a**) Depicted is a schematic example trial (blue target, DP and same-color cue). (**b**) Depicted are the mean RTs and standard errors of the mean (error bars) of all participants. Results are plotted separately for the short (left panel) and long SOA (right panel), for same color cues (solid line) and cues of a different color (dashed line).

The procedure was similar to the one used in Experiment 5 (see Figure 7a). One crucial change was that the placeholder rings remained on the screen for the whole trial, and the cue was an earlier color change of one of these rings rather than an abrupt-onset preceding the placeholders. In three different groups, participants searched either for red, green, or blue targets. For each participant, only one distractor color was used. The target was defined by a singleton color change to the predefined target color, and presented along with two disks changing to the same distractor color. For half of the participants, the placeholders were always black, for the other half they were always white. The cues equally likely shared the same color as the target—these were the top-down matching cues—or they changed to the distractor color—these were the non-matching cues. Again, both types of cues were presented equally often at the same and the different position as the target.

#### 2.6.3. Results

Trials with incorrect responses (1.3%) and RT outliers (further 1.4%) were again excluded from further analysis. 

#### 2.6.4. Subliminal Cueing Task

Results are depicted in Figure 7b. A repeated measurements ANOVA with the factors *cue position* (SP *vs.* DP), *cue color* (same *vs.* different color), and *SOA* (short *vs.* long) on mean correct RTs led to the following results. We found a significant main effect for cue color, *F*(1, 23) = 24.8, *p* < 0.001, η_p_^2^ = 0.52, reflecting that responses to different (or non-matching) color cues (RT = 392 ms) were generally faster than to same (or top-down matching) color cues (RT = 416 ms). The main effect for SOA *F*(1, 23) = 7.0, *p* < 0.05, η_p_^2^ = 0.24, confirmed that participants responded faster in the long SOA (RT = 392 ms) than the short SOA (RT = 416 ms). All other effects failed to reach significance.

#### 2.6.5. Visibility of Subliminal Cues and Its Correlation with the Cueing Effects

Participants in the final Experiment 6 were also unaware of the cues. As an objective measure of cue discrimination we calculated the sensitivity measure *d’* and the response criterion *c* for the target present trials. Mean *d’* (=1.9; range from −0.8 to 3.7) was significantly above chance-level (*p* < 0.001); mean *c* (=−0.1; range from −0.9 to 1.0) did not significantly differ from zero (*p* = 0.50).

**Figure A6:**
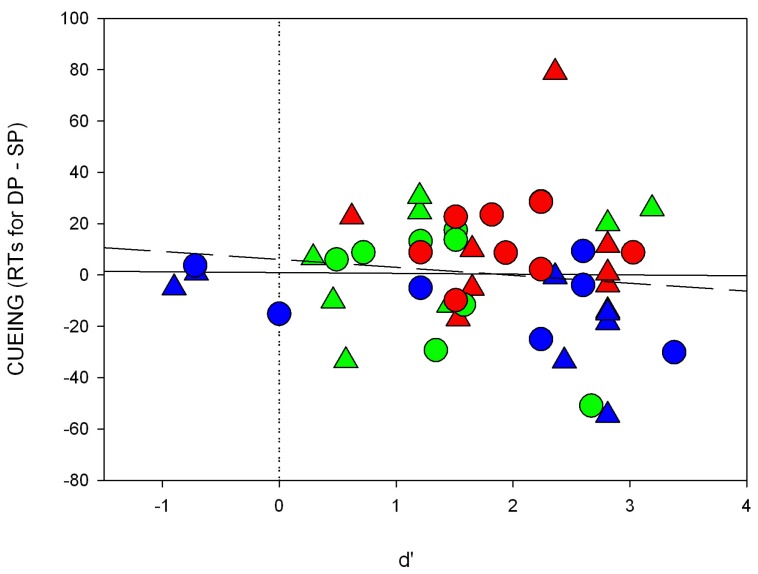
Individual scatter plots of the short SOA for Experiment 6. Shown are RT differences (cueing effects, *y*-axis) and the respective *d’*-value (*x*-axis) for each participant separately. Values are plotted separately for cues with the same (triangles; solid regression line) and a different color (circles; dashed regression line). Symbols are colored corresponding to the respective searched-for target color.

We calculated regressions of the RT differences between the SP and DP conditions as a function of discrimination (*d’*) for the short SOA (cueing effect, *i.e.*, RTs for DP–SP trials; see also Figure A6 of the Appendix for the results). For the short SOA, we found that the intercept was not significantly different from zero (*a* = 0.9, *p* = 0.91) and no significant correlation between discrimination and RT differences (beta = −0.03, *p* = 0.91). 

#### 2.6.6. Discussion

Here we tested, whether top-down-contingent capture for color can account for the lack of the attentional effects in Experiments 4 and 5. The main finding of the current Experiment 6 is that even under conditions where cue and target shared the searched-for color, the cues do not capture attention. This was indicated by the lack of a cueing effect in the short cue-target interval with similar (or top-down matching) cues and the absence of IOR in the long SOA. The results disconfirm that top-down contingent capture for color provided no better explanation of our results than the SC hypothesis. 

## 3. General Discussion

In the current study, we tested several crucial predictions of the hypothesis that the SC mediates unconscious attention effects of abrupt onsets [15,17]. In Experiment 1, we tested and confirmed that color cues and color targets created subliminal cueing effects. This was the case although the cue and target colors were not the same. For example, a subliminal blue cue captured attention, even if a black target was used. Likewise a subliminal black cue created a cueing effect where a blue target was searched-for. This result is in line with the SC hypothesis because the luminance of the color cues and color targets were only equated with the objective background luminance. As a consequence, different color sensitivities of the participants should have led to color-elicited contrast responses. These contrast responses to the color cues were evidently sufficient for the cueing effects because whether or not the contrasts of cues and targets were or were not the same was irrelevant for the cueing effect. The present subliminal cueing effect is therefore in line with the SC’s polarity-independent attentional effects of visible [26] and unconscious cues [27]. 

A second crucial prediction of the SC hypothesis was tested in Experiment 2. In this experiment, we investigated whether the attentional effect of the subliminal abrupt-onset cues was proportional to the contrast strength of the cues [36]. In line with this hypothesis stronger cueing effects were found for high-contrast cues compared to low-contrast cues (Experiment 2). 

A third critical prediction of the SC hypothesis was tested in Experiment 3. Prior studies showed that the SC response is larger for temporally compared to nasally presented onset stimuli [53]. This is also true of subliminal cues [43]. In line with prior findings [51] a naso-temporal asymmetry of the subliminal cueing effect was found in Experiment 3. 

We also reasoned that if the SC’s contrast-dependent response is responsible for the subliminal cueing effect, the contrast-elicited subliminal cueing effects should only be found when contrasts could be used to easily locate the target. After all, the participants have to find the target. Finding the target with the help of contrast should be more difficult if more than one stimulus elicits a contrast change at the time of the target. In this situation, different visual features all compete for the guidance of attention towards the relevant stimuli, and the feature that provides the easiest target-distractor discrimination and the strongest target-distractor difference should succeed in a competition for attentional guidance [37,38]. If it happens that the succeeding attention-guiding feature is processed outside of the SC, as would be the case with color [25], attentional guidance would be tipped away from the SC and towards the more sensitive feature channels of the visual system [39]. With color, these channels would lie in the parvocellular projection [25,41]. In this situation the SC should be shunted.

In line with this prediction, we found no subliminal cueing effects when a color target was presented together with color distractors, so that the color-elicited contrast differences between target and distractors were kept relatively small. The predicted lack of subliminal cueing was observed under these conditions when a color feature had to be used to find the target (Experiment 4), when a color difference could have been used to locate the target (Experiment 5), and even when the cue and the target shared the searched-for color.

Besides, we observed several unexpected results. First of all, cue discrimination was too high to pass a criterion of objective subliminality. This is in contrast to prior studies [15]. Yet, our participants were subjectively unaware of the cues in all experiments, and unawareness was also confirmed by a significant offset at the point of zero visibility of the regression predicting the cueing effect on the basis of discrimination [46]. 

A final unexpected observation was the similar strength of the cueing effects of colored and of black cues (Experiment 1). On the basis of the stronger objective contrast of the black cues than the color cues, an SC mediated cueing effect should have been stronger with the black cues than the color cues [26,36]; see also the present Experiments 2. Maybe this lack of a contrast-dependence of the cueing effect reflected strong subjective color-elicited contrast responses. We cannot reject this possibility because we did not quantify individual color-elicited contrast perception. Nevertheless, we also found attentional effects in line with this assumption of cueing effects proportional to the strength of the cue’s contrast (Experiment 2).

## 4. Conclusions

In the current study, we tested whether conflicting results on subliminal cueing that were reported in the literature can be explained by one hypothesis: If participants can make use of contrast information to find a target, a subliminal contrast cue captures attention via a color-insensitive part of the magnocellular pathway [15,17]. However, if color information is better suited to locate the targets than contrast, attention is guided by the parvocellular projection and the SC pathway is shunted [37,40] so that subliminal onset cues fail to capture attention [32,33]. Together, these assumptions emphasize the role of the SC for contrast-driven and largely color-independent subliminal attention.
